# Epithelial Cells and Fibroblasts from the Human Female Reproductive Tract Accumulate and Release TFV and TAF to Sustain Inhibition of HIV Infection of CD4+ T cells

**DOI:** 10.1038/s41598-018-38205-y

**Published:** 2019-02-12

**Authors:** Zheng Shen, Marta Rodriguez-Garcia, Mickey V. Patel, Jack Bodwell, Charles R. Wira

**Affiliations:** Department of Microbiology and Immunology, Geisel School of Medicine at Dartmouth, Lebanon, NH USA

## Abstract

Tenofovir (TFV) treatment of female reproductive tract (FRT) cells results in differential accumulation of intracellular Tenofovir diphosphate (TFV-DP) in different cell types, with greater concentrations in epithelial cells (100-fold) and fibroblasts (10-fold) than in CD4+ T cells. The possibility that TFV-DP accumulation and retention in epithelial cells and fibroblasts may alter TFV availability and protection of CD4+ T cells against HIV infection, prompted us to evaluate TFV and/or Tenofovir alafenamide (TAF) release from FRT cells. Endometrial, endocervical and ectocervical polarized epithelial cells and fibroblasts were pre-loaded with TFV or TAF, and secretions tested for their ability to inhibit HIV infection of activated blood CD4+ T cells. Epithelial cell basolateral secretions (1, 2 and 3 days post-loading), but not apical secretions, suppressed HIV infection of CD4+ T cells, as did secretions from pre-loaded fibroblasts from each site. Intracellular TFV-DP levels in epithelial cells following preloading with TFV or TAF correlated directly with ARV protection of CD4+ T cells from HIV infection. When added apically to epithelial cells, TFV/TAF was released basolaterally, in part through Multidrug Resistant Protein transporters, taken up by fibroblasts and released into secretions to partially protect CD4+ T cells. These findings demonstrate that epithelial cells and fibroblasts release TFV/TAF for use by CD4+ T cells and suggest that the tissue environment plays a major role in the sustained protection against HIV infection.

## Introduction

Half of the people infected with HIV worldwide are women^1^. In endemic areas like Sub-Sharan Africa however, women are at disproportionate increased risk for HIV acquisition compared to men, and HIV is the main cause of death for reproductive age women^2^. Sexual transmission is the main route for HIV acquisition in women, therefore, preventive strategies in women need to be effective in the female reproductive tract (FRT). The immune system in the FRT has the dual role of protecting against infections while allowing pregnancy to occur^3^. To this end, immune cells in the FRT are tightly regulated by sex hormones and the tissue environment, which control immune cell distribution and function^3–10^.

Central to the strategy of preventing the sexual transmission of HIV to women is the use of pre-exposure prophylaxis (PrEP), in which antiretrovirals (ARVs) such as Tenofovir (TFV) are delivered topically into the vagina or taken orally as tenofovir disoproxil fumarate and emtricitabine (TDF/FTC; Truvada). Oral PrEP^11^ was shown in several trials to protect against HIV-1 infection in heterosexual men and women^12–14^. In contrast, only one trial (CAPRISA 004) using topical TFV applied in the vagina has shown significant protection against HIV acquisition in women, while several other trials involving only women, using topical or oral PrEP (Fem PrEP, FACTS, and VOICE) have shown no protective effect^15–17^. Beyond compliance, the success or failure of ARVs depends on effective concentrations of ARVs being achieved and maintained in those tissue cells (CD4+ T cells and macrophages) susceptible to HIV-1 infection.

TFV and its prodrug tenofovir alafenamide (TAF) are HIV nucleoside analog reverse transcriptase inhibitors that act via their integration into nascent viral DNA to prevent transcription of the viral RNA into viral DNA, a key early step in the HIV lifecycle. TFV and TAF, differ in their ability to enter cells. TFV with its inherent negative charge is poorly taken up by cells and is dependent on limited diffusion as well as energy dependent transporters^18–21^. TAF, due to its neutral charge, readily diffuses into the cell, although transporters may also be involved in cell entry^22^. Thus TAF achieves similar protection against HIV infection at concentrations ~300 fold lower than TFV^7^. Intracellular TAF is readily converted to TFV via the actions of Cathepsin A. Once in the cell, TFV is converted into TFV-diphosphate (TFV-DP) through two sequential phosphorylation reactions^23^. It is TFV-DP, the active metabolite of TFV and TAF, which interferes with viral replication.

Previous studies by us evaluated the intracellular concentrations of TFV-DP (the active form of TFV) in purified immune and non-immune cells from the upper and lower human FRT^24^. We found that concentrations of TFV-DP were 100-fold higher in epithelial cells and 10-fold higher in fibroblasts when compared to CD4+ T cells and macrophages. In other studies, the distribution of TFV-DP was analyzed using combined confocal Raman spectroscopy (CRS) and optical coherence tomography (OCT) to measure the distribution of TFV in intact porcine vaginal tissues^25,26^. Measured with sub-100-micron spatial resolution, the concentration of TFV following topical application was greatest in the epithelium and rapidly diminished deeper in the stroma. Taken together, these findings indicate a cell-specific distribution of TFV-DP in the reproductive tract and demonstrate that tissue biopsy concentrations may not reflect the physiologically-relevant concentrations of an ARV needed to prevent the sexual transmission of HIV. The recognition that ARVs are not uniformly distributed between cells in the reproductive tract emphasizes the need to understand the role of the tissue environment in modulating protection and susceptibility to HIV infection.

When TFV is applied topically into the vagina, it first enters epithelial cells at the apical (luminal) surface after which it is released basolaterally into the subepithelial (stromal) compartment, possibly through several mechanisms, including passive diffusion and/ or transporters, such as breast cancer resistant protein (BCRP) and P-glycoprotein (P-gp) or Multidrug Resistant Proteins (MRP)^27–29^. In contrast, following oral administration, TFV reaches the subepithelial stroma of the reproductive tract through the blood stream^30^. Important differences have been described in TFV-DP vaginal levels after topical and oral administration of TFV^31,32^. Whether epithelial cells and fibroblasts act as a sink for TFV-DP to compromise protection, or release TFV slowly after initial uptake to maintain protection in the genital mucosa is unknown.

Using polarized primary epithelial cells and fibroblasts from the endometrium (EM), endocervix (CX) and ectocervix (ECX), we examined whether TFV- and/or TAF-derived intracellular accumulation of TFV-DP enhances or inhibits TFV-mediated protection against HIV by interfering with availability to CD4+ T cells. When epithelial cells were pre-loaded with TFV or TAF, we found that both ARVs were released basolaterally for at least 3 days at concentrations that provide partial protection of CD4+ T cells from HIV infection, and that ARV release was partially due to Multidrug Resistant Protein (MRP) transporters. These findings demonstrate that epithelial cells and fibroblasts contribute to mucosal protection against HIV throughout the FRT by acting as an ARV reservoir that extends partial protection following ARV administration.

## Materials and Methods

### Ethics statement

All human subject work was carried out with the approval of the Dartmouth College Institutional Review Board. Approval to use tissues was previously obtained from the Committee for the Protection of Human Subjects (CPHS), and with written informed consent obtained from the patient before surgery. All samples were anonymized, and all investigations were conducted according to the principles expressed in the Declaration of Helsinki.

### Source of tissue and blood

Human FRT tissues were obtained immediately following surgery from women who had undergone hysterectomies at Dartmouth-Hitchcock Medical Center (Lebanon, NH). Tissues from the endometrium (EM), endocervix (CX) and ectocervix (ECX) were collected from patients with benign conditions such as fibroids and prolapse (age from 26 to 61 years old). Tissue samples were distal from the sites of pathology and were without pathological lesions as determined by a pathologist. Blood donors were anonymous, no information regarding age or hormonal status was available and only female donors were used in this study.

### Preparation of blood CD4+ T cells

Blood from women was obtained from our IRB-approved Blood Donor Program at Dartmouth-Hitchcock Medical Center. CD4+ T cells were purified with the CD4+ T cell isolation kit (Miltenyi Biotech) from frozen peripheral blood mononuclear cells (PBMC) as described before^5,8^. Purified blood CD4+ T cells were activated *in vitro* using X-vivo 15 media (Lonza, Walkersville, MD) with Phenol Red plus Phytohemagglutinin (PHA, 2.5 µg/ml; Sigma, St Louis, MO) and IL-2 (50 U/ml, AIDS Research and Reference Reagent Program, Division of AIDS, NIAID, NIH: Human rIL-2 from Dr. Maurice Gately, Hoffmann- La Roche Inc.) for 24 hr as described previously^5,8^. Activated CD4+ T cells were plated at a density of 1 × 10^5^ cells per well in round-bottom 96-well culture plates (Corning, Corning, NY) in 0.1 ml of Immune cell media consisting of X-vivo 15 Media supplemented with Phenol Red and 10% human AB serum (Valley Biomedical, Winchester, VA) prior to treatment.

### Tissue processing

Tissues were rinsed with HBSS (Hanks balanced salt solution) supplemented with Phenol Red, 100 U/ml penicillin, 100 µg/ml streptomycin (all Thermo Scientific Hyclone, Logan, UT), and 0.35 mg/ml NaCO_3_ (Thermo Fisher Scientific, Pittsburgh, PA) and processed as previously described^7,8,33,34^. Tissues were then minced under sterile conditions into 1-2 mm fragments and digested at 37 °C for 1 hr using a mixture containing (final concentrations): 0.05% collagenase type IV (Sigma-Aldrich, St. Louis, MO) and 0.01% DNase (Worthington Biochemical, Lakewood, NJ) in HBSS (Invitrogen Life Technologies, Grand Island, NY). Type IV collagenase was selected based on preliminary studies to ensure non-cleavage of surface markers^4,6^. After digestion, cells were dispersed through a 250-µm nylon mesh screen (Small Parts, Miami Lakes, FL), washed, and resuspended in complete media consisting of DMEM/F12 medium without Phenol Red, supplemented with 10 mM HEPES (both GIBCO, Life Technologies, Grand Island, NY), 100 µg/ml primocin (InvivoGen, San Diego, CA), 2 mM L-glutamine, 2.5% heat-inactivated defined fetal Bovine Serum (FBS) (both from Thermo Scientific Hyclone) and 2.5% NuSerum (BD Biosciences, Bedford, MA). Epithelial cell sheets were separated from stromal cells by filtration through a 20-µm mesh filter (Small Parts). Epithelial cell sheets were retained on the filter, while stromal cells passed through.

### Isolation and culture of FRT epithelial cells and stromal fibroblasts

Epithelial cell sheets were recovered by rinsing and backwashing the filter with complete medium, centrifuged at 500 g for 5 min and analyzed for cell number and viability as previously described^7,24,34^. To establish a cell culture system of polarized human FRT epithelial cells with both apical and basolateral compartments, FRT epithelial cells were cultured in Matrigel matrix (BD Biosciences) coated Falcon cell culture inserts in 24-well companion culture plates (Fisher Scientific). Apical and basolateral compartments contained 300 and 500 µl of complete medium, respectively, which was changed every 2 days. Tight junction formation of epithelial cell monolayers from EM and CX was assessed by periodically measuring transepithelial resistance (TER) using an EVOM electrode and Voltammeter (World Precision Instruments, Sarasota, FL), as described previously^35^. To keep the culture conditions similar, the same procedure was followed for culturing squamous ECX epithelial cells, which do not polarize.

Stromal fibroblasts were isolated as previously described^7,34,36^. Briefly, following removal of epithelial sheets, the flow-through containing stromal fibroblasts and immune cells was collected, centrifuged at 500 × *g* for 10 min, resuspended, cell number and viability determined. Freshly isolated stromal fibroblasts and immune cells were incubated in complete medium in a T75 cell culture flask (Fisher Scientific, Pittsburgh, PA). Media was replaced every 48 hr to remove non-adherent cells. Once cells reached confluence, they were trypsinized and plated at a concentration of 1 × 10^5^ cells/well in 24-well culture plates (Fisher Scientific) for at least 48 hr prior to treatment.

### TFV and TAF preparation

TFV in powder form was obtained from AIDS Research and Reference Reagent Program (NIH AIDS Reagent Program, Division of AIDS, NIAID, NIH: Tenofovir, catalog number 10199). A stock concentration of TFV 5 mg/ml was prepared by adding 1 ml of PBS to 5 mg of TFV powder, before being diluted in stripped media to the appropriate working concentration^7,8,24,37^. TAF was kindly supplied by Gilead Sciences Inc. (Foster City, CA) and was dissolved in PBS at 10 mM, sterilely filtered (0.2 um) and the concentration checked by absorbance using a molar extinction coefficient of 11690 at 260 nm^7,8^. Subsequent dilutions of TFV and TAF were made in complete media to the appropriate working concentrations.

### Intracellular TFV-DP measurement

TFV or TAF were added to polarized epithelial cells both apically (lumen) and basolaterally (tissue) compartments for 24 hr. For some experiments, TFV and TAF were added only apically as indicated in the Results. After treatment, cells were washed, harvested and lysed in 300 µl of 70% methanol, and stored immediately at −80 °C prior to TFV-DP evaluation as previously described^8,24^. Intracellular TFV-DP concentrations were measured by liquid chromatography with tandem mass spectrometry (LC-MS/MS) and normalized values to fmol/million cells based on the number of cells per sample^32^.

### Collection of secretions from epithelial cells and fibroblasts

TFV or TAF were added to polarized epithelial cells both apically (lumen) and basolaterally (tissue) compartments for 24 hr, or only apically, as indicated. The time of measurement of TFV-DP used in this study and in our previous research^7,8,24^, was based on the initial clinical trial (CAPRISA 004) which used topical TFV applied in the vagina and reported significant protection against HIV acquisition in women when taken between 24 hr prior to sexual intercourse to 24 hr after sex^15^. Following incubation, cells were washed in media 3 times to remove extracellular TFV or TAF, after which epithelial cell inserts were transferred into new wells and fresh media was added to both the apical and basolateral compartments. Conditioned media (CM) were recovered from apical and basolateral compartments of cell inserts after 24 hr. Similarly, TFV or TAF was added to stromal fibroblasts grown to confluence in 24-well plates for 24 hr, after which they were washed repeatedly prior to the addition of fresh media to collect CM at 24 hr. For epithelial cell time course experiments, media in each compartment was replaced at 24 hr intervals (1, 2, and 3 days), CM was collected at each time point. To evaluate the interaction between epithelial cells and fibroblasts, polarized epithelial cell inserts were transferred to 24-well plates containing confluent subject-paired fibroblast cultures. TFV or TAF were added to polarized epithelial cells apically for 24 hr, after which basolateral CM was collected. At the end of this incubation, cell inserts and incubation chambers +/− fibroblasts were washed thoroughly both apically and basolaterally so that both epithelial cells and fibroblasts were free of any extracellular TFV or TAF. Cells were then divided in to 2 separate groups consisting of EC inserts transferred to new wells (no fibroblasts), and fibroblasts in the lower chamber alone (no EC inserts). In each of these combinations, fresh media lacking ARVs was added and cells incubated for an additional 24 hr after which conditioned media (containing released intracellular ARVs) was recovered and analyzed for anti-HIV activity.

### Inhibition of Multidrug Resistance-associated Protein (MRPs)

To evaluate the role of MRPs in ARV movement, epithelial cells were incubated with TFV or TAF for 20 hr, followed by the addition of human MRP-specific inhibitor MK571 (Sigma-Aldrich, St Louis, MO) for 4 hr. MK571 was added in 250 µl of complete media to both the apical and basolateral compartments and was present in the fresh media following cell rinsing and incubation. CM was collected from apical and basolateral compartments of cell inserts after 24 hr. CM was centrifuged at 10,000 g and stored at −80 °C until HIV infection assay. Untreated control cells were donor-matched to treated cells and processed in parallel at the same time. Cell viability was tested after treatment using the CellTiter 96 AQ_ueous_ One Solution cell proliferation assay (Promega, Madison, WI, USA) and trypan blue staining (HyClone Laboratories, Inc., Logan, UT) as described before^7,8^. All samples were assayed blind without any information provided as to cell origin and treatment.

### HIV-infection

Activated blood CD4+ T cells were infected as previously described with minor modifications^5,8^. Stock for HIV-BaL (R5) was obtained through the AIDS Research and Reference Reagent Program, Division of AIDS, NIAID, NIH, from Dr. Suzanne Gartner, Dr. Mikulas Popovic and Dr. Robert Gallo^38^. Briefly, activated blood CD4+ T cells were incubated with apical and basolateral epithelial cell or fibroblast CM for 24 hr and then washed with PBS prior to HIV infection. After washing, cells were incubated with HIV-BaL for 2 hr at an MOI of 0.1 and then washed to remove residual virus. Fresh 0.2 ml IL-2 supplemented immune cell media was added to each well and cells were incubated for 5 days, with half of the media from each well collected and replaced with fresh media on day 3. Released p24 in the culture media on day 5 was measured by p24 enzyme-linked immunosorbent assay (Advanced Bioscience laboratories, Rockville, MD) following the manufacturer’s recommendations.

### RNA isolation and quantitative RT-PCR analysis

Real-time reverse transcription-polymerase chain reaction (RT-PCR) was done with a two-step protocol as described previously^34,36^. Total RNA was isolated from epithelial cells using RNeasy reagent (Qiagen, Valencia, CA) and QIAshredder columns according to the manufacturer’s recommendations (Qiagen), and purified by elution through RNeasy columns (Qiagen) with on-column DNase digestion using the RNase-Free DNase set (Qiagen). For each specimen, 400 ng of total RNA was reverse-transcribed using the iScript complementary DNA (cDNA) synthesis kit (Bio-Rad, Hercules, CA) according to the manufacturer’s recommendations. Relative mRNA expression levels of genes of interest were measured using the 5′ fluorogenic nuclease assay in real-time quantitative PCR using TaqMan chemistry on the ABI 7300 Prism real-time PCR instrument (Applied Biosystems, Foster City, CA). The 5 members from human multidrug resistance-associated protein family (MRP1, 3, 4, 5 and 6) (ID nos. Hs01561483_m1, Hs00978452_m1, Hs00988721_m1, Hs00981089_m1, Hs01077866_m1, respectively) and β-actin (4333762 F) primer/MGB probe sets were obtained from Applied Biosystems assays-on-demand. The 4 epithelial cell tight junction genes analyzed included TJP1 (Zona Occluden 1) (ID nos. Hs01551861_m1), OCLN (Occludin) (ID nos. Hs00170162_m1), CLDN2 (ID nos. Hs00252666_s1) and 4 (ID nos. Hs00533616_s1) (Claudin 2 and 4). PCR was conducted using the following cycle parameters: 12 min at 95 °C for one cycle, followed by 40 cycles of 20 seconds at 95 °C and 1 min at 60 °C. Analysis was conducted using the sequence detection software supplied with the ABI 7300. The software calculates the threshold cycle (C_t_) for each reaction and this was used to quantify the amount of starting template in the reaction. The C_t_ values for each set of duplicate reactions were averaged for all subsequent calculations. A difference in C_t_ values (ΔC_t_) was calculated for each gene by taking the mean C_t_ of each gene of interest and subtracting the mean C_t_ for the housekeeping gene β-actin for each cDNA sample. The relative expression level of each gene was calculated using the formula 2^−ΔCt^.

### Statistics

Data analysis was performed using the GraphPad Prism 5.0 (GraphPad Software, San Diego, CA). A two-sided P value < 0.05 was considered statistically significant. Comparison of three or more groups was performed applying Kruskal-Wallis test for non-matched samples or Friedman test for matched samples, followed by Dunns-post test for multiple comparison correction. Comparison studies of HIV infections in the absence vs presence of inhibitor MK571 or accumulation of TFV-DP from apical and basolateral treatment and time course studies were analyzed using two-way ANOVA with Bonferroni post-test for multiple comparison correction.

## Results

### Conversion of TFV and TAF into intracellular TFV-DP by Endometrial Epithelial Cells

**Figure 1:**
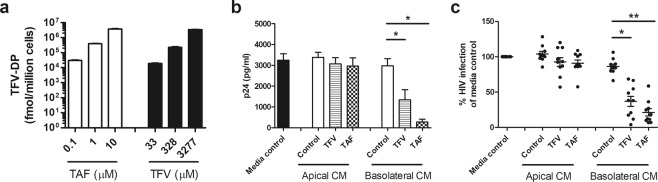
Evaluation of the protective effect of secretions from endometrial epithelial cells following preincubation with TFV or TAF on HIV infection of blood CD4+ T cells. (**a**) Dose-dependent increase in intracellular TFV-DP levels following treatment of purified polarized endometrial (EM) epithelial cells with TAF (white bars) or TFV (black bars) for 24 hr. Bars and horizontal lines represent mean and SEM respectively from triplicate cultures of cells from a representative patient. Values are expressed as fmol/million cells. (**b**,**c**) Levels of HIV infection (released p24) in CD4+ T cells after incubation with apical and basolateral CM from EM epithelial cells pre-treated with TFV (3277 µM) or TAF (10 µM) as described in Methods. (**b**) Representative example of secreted p24 levels from a single patient run in quadruplicate. Columns and horizontal lines represent the mean and SEM respectively. (**c**) Secreted p24 values are normalized to the infection of CD4+ T cells in the absence of CM (media control) which is set to 100%. Each circle represents an individual patient (n = 10) and horizontal lines represent the mean and SEM. Blood CD4+ T cells were isolated from 4 donors. *p < 0.05. **p < 0.01.

In order to compare the protective effects of TFV and TAF, studies were undertaken to ensure that comparable concentrations of TFV-DP were present in polarized Endometrial (EM) epithelial cells following incubation with each ARV. These studies were carried out based on the recognition that TAF is administered clinically at lower doses than TFV and that intracellular conversion of TAF into TFV-DP is much more efficient than TFV^22^. EM epithelial cells were treated with different doses of TFV or TAF for 24 hr and intracellular levels TFV-DP were determined^7^. Using this data as a standard curve, we then calculated TFV and TAF doses that would result in equal amounts of intracellular TFV-DP to compare both drugs side by side. As seen in Fig. 1a and presented elsewhere^7^, we predetermined the dose for each ARV that would give the same intracellular concentration of TFV-DP. Incubation with TFV at 3 concentrations (33, 328, and 3277 μM) and TAF (0.1, 1, and 10 μM) resulted in equivalent amounts of intracellular TFV-DP. Exposure for 24 hr was chosen based on our previous publications^7,24^ and the initial clinical trial (CAPRISA 004) that reported significant protection against HIV acquisition in women that applied topical TFV in the vagina 24 hr prior to sexual intercourse^15,39^. The findings presented here provide the foundation for comparisons in the following sections that measure the effectiveness of ARVs released into secretions for their ability to protect target cells from HIV infection.

### Basolateral secretions from polarized endometrial epithelial cells incubated with TFV or TAF protect CD4+ T cells from HIV infection

Based on our findings in Fig. 1a, the concentrations of TFV (3277 μM) and TAF (10 μM) were selected to evaluate whether preloaded polarized endometrial epithelial cells release their products in a way that confers protection against HIV infection. After incubation with TFV or TAF for 24 hr followed by extensive washing to remove excess ARVs, epithelial cells were incubated for an additional 24 hr in fresh media prior to recovery of apical and basolateral conditioned media (CM). This CM should contain any TFV or TAF released by epithelial cells. To test if released TFV from epithelial cells would confer protection, activated blood CD4+ T cells were incubated with apical or basolateral CM for 24 hr prior to *in vitro* HIV infection as detailed in methods. As shown in Fig. 1b, in a representative experiment, basolateral CM from TFV and TAF treated polarized epithelial cells, but not apical CM, significantly inhibited HIV infection of CD4+ T cells. As seen in Fig. 1c, when 10 patients were evaluated, we found that following epithelial preloading with TFV or TAF, basolaterally secreted CM protected against HIV infection (mean inhibition = 60% and 80% respectively). In all of these experiments, apical secretions showed no evidence of protection.

**Figure 2:**
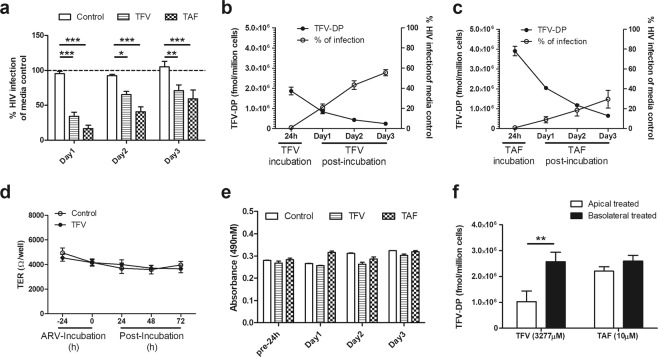
Protective anti-HIV effect of basolateral secretions from endometrial (EM) epithelial cells following treatment with TFV or TAF correlates with epithelial levels of intracellular TFV-DP. (**a**) HIV infection levels in CD4+ T cells after incubation with CM from EM epithelial cells pre-treated with TFV or TAF. EM epithelial cells were pre-treated with either TFV (3277 µM) or TAF (10 µM) for 24 hr, after which ARVs were washed out of cell culture. Cells were then incubated with fresh media that was replaced daily for an initial 24 hr (Day 1), a second 24 hr period (Day 2), and a third 24 hr period (Day 3) after which basolateral conditioned media (CM) was collected. Activated CD4+ T cells were treated for 24 hr with CM recovered at Day 1, 2, and 3. Following washout of CM, CD4+ T cells were infected after which secreted p24 levels measured by p24 ELISA after 5 days as described in Methods. Data are normalized to the infection of CD4+ T cells in the absence of CM (media control) which is set to 100% (dashed line). (n = 4 individual patients). Columns and horizontal lines represent the mean and SEM respectively. *p < 0.05. **p < 0.01. ***p < 0.001. (**b** and **c**) Intracellular TFV-DP levels in EM epithelial cells are inversely related to HIV infection of CD4+ T cells treated with EM epithelial CM. Intracellular TFV-DP levels in EM epithelial cells were measured by LC-MS/MS following TFV (**b**) and TAF (**c**) treatment (24 hr), followed by incubation for 24 hr intervals with fresh media collection on Day 1, Day 2, and Day 3. CM was collected at each time point along with cell recovery to measure HIV infection as described in Methods. Circles and horizontal lines represent the mean and SEM from triplicates in a single representative patient. (**d**) Time course of the lack of an effect on transepithelial resistance (TER) of polarized epithelial cells treated with TFV (3277 µM) for 24 hr after which ARV was washed out of cell culture. Cells were then incubated with fresh media that was replaced daily for 3 days. (**e**) Lack of an effect of TFV and TAF on epithelial cell viability. Polarized EM epithelial cells were apically and basolaterally treated with TFV (3277 µM) or TAF (10 µM) for 24 hr prior to washout and measurement of viability on days 1, 2 and 3. Cell viability was tested with CellTiter 96 AQ_ueous_ One Solution cell proliferation assay. The bars represent the mean and SEM of triplicate cell inserts. (**f**) Intracellular accumulation of TFV-DP on endometrial epithelial cells following apical or basolateral incubation with TFV or TAF. Intracellular TFV-DP levels were measured by LC-MS/MS in purified polarized endometrial epithelial cells treated with TFV (3277 µM) or TAF (10 µM) apical or basolateral for 24 hr. Values are expressed as fmol/million cells. The bars represent the mean and SEM from 3 patients. **p < 0.01.

To determine whether protection by basolateral secretions persisted beyond 24 hr, CM was collected and replaced with fresh media daily for 3 days. As seen in Fig. 2a, protection by TFV and TAF in basolateral secretions persisted for 3 days in that each conferred partial protection against HIV infection of CD4+ T cells. By day 3, protection relative to media controls was lower than that measured on day 1. Despite this decline, significant inhibition of infection by HIV of activated CD4+ T cells was observed in 4/4 experiments. This progressive decline in HIV-protection correlated with a slow decline in intracellular TFV-DP levels in the epithelial cells (Fig. 2b,c). Interestingly, combining the data for the percent inhibition of CD4+ T cells to TFV-DP levels for both TFV- and TAF-treated epithelial cells yielded a smooth curve showing a 50 percent inhibition corresponding to a TFV-DP concentration of ~340,000 fmol/million cells (Suppl. Fig. 1a). This correlation between epithelial cell TFV-DP levels and protection to CD4+ T cells from HIV supports our hypothesis that protection is due to basolateral release of TFV and TAF into the basolateral secretions of epithelial cells. At no time was protection by ARVs evident in apical CM over the course of these experiments, demonstrating that EC-release of TFV is selective towards the subepithelial (tissue) compartment.

Recognizing that cell integrity is essential, we measured transepithelial resistance (TER) both prior to the addition of TFV and following its addition and wash out both apically and basolaterally for 3 days in culture. As seen in Fig. 2d, when TER was measured daily TFV treatment had no significant effect on TER, indicating that cell and barrier integrity was maintained throughout the course of our experiments. In parallel, we measured the impact of ARVs on tight junction gene expression and found that TFV had no effect on TJP1, OCLD, CLDN2 and CLDN4 mRNA expression in polarized epithelial cells (Suppl. Fig. 1b). As a further indicator of cell viability, in separate studies, polarized EM epithelial cells were pretreated with TFV (3277 μM) and TAF (10 μM) for 24 hr, prior to measuring viability at 24 hr intervals for 3 days. As shown in Fig. 2e, no changes in viability were found for either ARV. These data demonstrate that the protective ARV levels found in the basolateral compartment in our studies are not due to ARV leakage through epithelial damage.

Recognizing that ARVs following preloading are preferentially released basolaterally, we investigated whether cell uptake of TFV and TAF also exhibited polarity. As seen in Fig. 2f, we found that directional application (apical vs basolateral) of TFV and/or TAF influenced TFV-DP concentrations within epithelial cells. When measured after 24 hr incubation, epithelial cells incubated basolaterally with TFV had 2–3 fold more TFV-DP than did matched cells incubated apically with TFV. In contrast, TAF applied to either the apical or basolateral surface of polarized epithelial cells resulted in comparable intracellular concentrations of TFV-DP.

Overall, these findings indicate that epithelial cells preloaded with either TFV or TAF gradually release ARVs basolaterally in a way that confers partial tissue protection of CD4+ T cells that persists for days.

### Endocervical and ectocervical basolateral secretions from ARV-treated polarized epithelial cells protect against HIV infection

**Figure 3:**
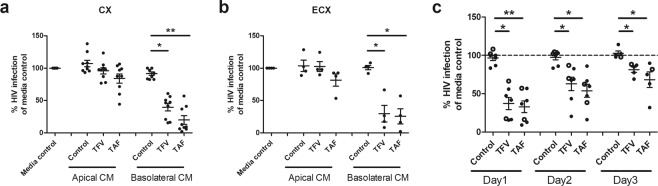
Secretions from endocervical and ectocervical epithelial cells treated with TFV or TAF inhibit HIV infection of CD4+ T cells. Apical and basolateral conditioned media (CM) were collected from endocervix (CX) and ectocervix (ECX) epithelial cells pre-treated with TFV (3277 µM) or TAF (10 µM) for 24 hr. CM were collected 24 hr post ARV washout and incubation with fresh media; CM was incubated with activated CD4+ T cells prior to HIV infection. Secreted p24 levels in the culture media after 5 days of infection were measured by p24 ELISA as described in Methods. Data are normalized to the infection of CD4+ T cells in the absence of CM (media control) which is set to 100% using **(a)** CM from CX epithelial cells from 9 patients, **(b)** CM from ECX epithelial cells from 4 patients. **(c)** Time course of HIV protection of CD4+ T cells by basolateral CM from CX (dark circles) and ECX (open circles) epithelial cells pre-treated with either ARVs for 24 hr prior to wash out. Cells were then incubated with fresh media that was replaced each day for 3 days, basolateral CM was collected daily. Activated CD4+ T cells were treated for 24 hr with CM. Following washout of CM, CD4+ T cells were infected after which secreted p24 levels measured by p24 ELISA as described in Methods. Data are normalized to the infection of CD4+ T cells in the absence of CM (media control) which is set to 100% (dashed line). (n = 7 individual patients). Columns and horizontal lines represent the mean and SEM respectively. *p < 0.05. **p < 0.01.

The possibility that the release pattern of ARVs was unique to endometrial epithelial cells prompted us to ask if epithelial cells from other sites in the FRT might share a similar pattern of ARV release. Purified endocervical (CX) and ectocervical (ECX) epithelial cells were grown to confluence, incubated with TFV or TAF for 24 hr and washed to remove excess ARVs after which CM was collected for 24 hr. When cells were preloaded with TFV or TAF, basolateral CM from CX (Fig. 3a) and ECX (Fig. 3b) epithelial cells protected against HIV infection of CD4+ T cells. Importantly, similar to EM epithelial cells, apical CM from CX and ECX cells had no protective effect against HIV infection. In other studies, we found that basolateral secretions collected at 24 hr intervals (days 1, 2 and 3) also protected against HIV infection, similar to that seen with EM epithelial cells (Fig. 3c). Therefore, EM, CX and ECX epithelial cells shared a pattern of TFV and TAF-derived release that was gradual and primarily basolateral towards the underlying stroma.

### Stromal fibroblasts from the FRT are a reservoir for gradual ARV release

**Figure 4:**
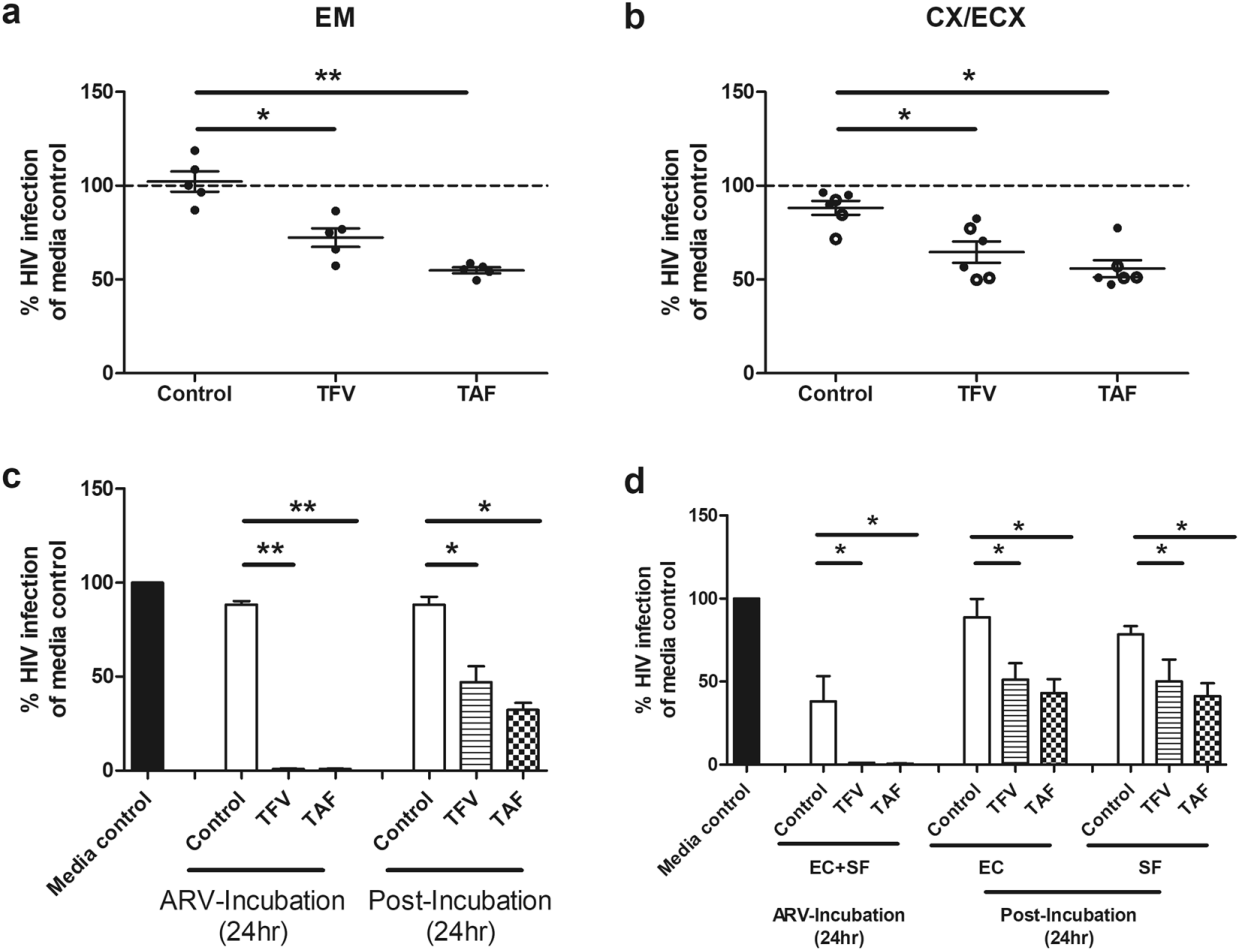
Effect of fibroblasts and endometrial (EM) epithelial cells in decreased HIV infection of CD4+ T cells. Conditioned media (CM) was collected from **(a)** endometrium (EM), **(b)** CX/ECX fibroblasts pre-treated with TFV (3277 µM) or TAF (10 µM) for 24 hr. CM were collected 24 hr post ARV washout and incubation with fresh media; CM was incubated with activated CD4+ T cells prior to HIV infection. Secreted p24 levels in the culture media after 5 days of infection were measured by p24 ELISA as described in Methods. CM was collected from EM fibroblasts of 5 patients while CM from CX (dark circle) and ECX (open circle) fibroblasts was from 3 matched patients. Each circle represents an individual patient. Data are normalized in **(a**,**b)** to the infection of CD4+ T cells in the absence of CM (media control) and set to 100%. Each circle represents a different patient. Blood CD4+ T cells were isolated from 4 donors. Horizontal lines represent the mean and SEM respectively. *p < 0.05. **p < 0.01. **(c)** Polarized EM epithelial cells were apically treated with TFV or TAF for 24 hr, after which basolateral CM was immediately collected (ARV-Incubation). Following rinsing, cells were incubated with fresh media (no TFV or TAF) for an additional 24 hr prior to CM collection (Post-Incubation 24 hr). Activated CD4+ T cells from blood were incubated with CM for 24 hr and washed and infected with HIV for 2 hr. Secreted viral p24 levels were measured after 5 days of infection (Methods). Data are normalized to the infection of CD4+ T cells in the absence of CM (media control) which is set to 100%. Bars represent EM tissues from 3 patients. **(d)** Effect of epithelial cells and fibroblasts on prevention of HIV infection. Polarized EM epithelial cells grown in the upper chamber of cell inserts were treated apically with TFV (3277 µM) or TAF (10 µM) for 24 hr in the presence of confluent fibroblasts (SF) from the same donor grown in the lower chamber (no cell contact). Following incubation, epithelial cells and stromal fibroblasts (EC + SF), basolateral CM was collected for analysis (ARV-Incubation). Following extensive washing to remove extracellular ARVs (Post-Incubation), epithelial cells alone (EC) and stromal fibroblasts alone (SF) were incubated in fresh media for 24 hr, after which CM were collected. CD4+ T cell protection against HIV infection for each CM was measured as described in Methods. Data are normalized to the infection of CD4+ T cells in the absence of CM (media control). Bars represent 4 patients. Blood CD4+ T cells were isolated from 2 donors. Column and horizontal lines represents the mean and SEM respectively. *p < 0.05. **p < 0.01.

Our previous studies indicated that in addition to epithelial cells, stromal fibroblasts from the FRT also concentrate intracellular TFV-DP approximately 10 fold higher than FRT CD4+ T cells^24^. To determine whether fibroblast secretions from the EM, CX and ECX were capable of protecting CD4+ T cells from HIV infection, fibroblasts grown to confluence in 24 well plates were incubated with either TFV (3277 μM) or TAF (10 μM) for 24 hr. Following repeated washes, cells were incubated for an additional 24 hr in fresh media prior to collection of CM for analysis. As shown in Fig. 4a,b, CM from EM as well as CX and ECX fibroblasts preloaded with TFV and TAF were capable of partially protecting activated CD4+ T cells from infection by HIV (30–50% protection).

### Protection by basolateral secretions from polarized EM epithelial cells incubated apically with ARVs

In topical Pre-exposure prophylaxis studies, TFV is deposited in the vagina at the epithelial cell surface after which it moves upstream into the ECX, CX and EM and into the underlying stroma containing CD4+ T cells^28,32^. To mimic the movement of ARVs from the luminal surface, polarized epithelial cells were treated apically with TFV or TAF followed by basolateral CM collection after 24 hr incubation. Following multiple washes of epithelial cells, cells were incubated with fresh media for an additional 24 hr after which CM was collected. As seen in Fig. 4c, when CM from the initial incubation with TFV or TAF was evaluated, complete protection of CD4+ T cells from HIV infection was observed (Fig. 4c; ARV-Incubation). Additionally, CM collected 24 hr after washout conferred partial protection (Fig. 4c; ARV-Post-Incubation), demonstrating continued basolateral release of ARVs by epithelial cells following cell washing of initial ARV input. These findings indicate that under conditions of apical exposure, ARVs continue to be released basolaterally after the apical source of ARV is removed.

To further mimic the interactions of ARVs with the cells present below the epithelium, we apically treated polarized epithelial cells with TFV or TAF for 24 hr during which stromal fibroblasts were present beneath but not in contact with epithelial cells (Fig. 4d). We found that basolateral CM collected during the first 24 hr from epithelial cells and stromal fibroblasts is able to fully protect CD4+ T cells from infection. Following repeated washings, epithelial cells and stromal fibroblasts were incubated separately (Post-incubation) for an additional 24 hr in fresh media. Under these conditions, both epithelial cells and fibroblasts released ARVs in sufficient concentrations to partially protect CD4+ T cells from HIV infection. This study demonstrates that under conditions of apical epithelial cell exposure, ARVs move to the basolateral compartment to be taken up by fibroblasts for subsequent release and protection of CD4+ T cells. These studies suggest that *in situ*, both epithelial cells and fibroblasts release ARVs that contribute to the protection of CD4+ T cells in stromal tissues of the FRT.

### Multidrug resistance-associated proteins (MRP) are partially responsible for selective transport of ARVs into the subepithelial compartment

**Figure 5:**
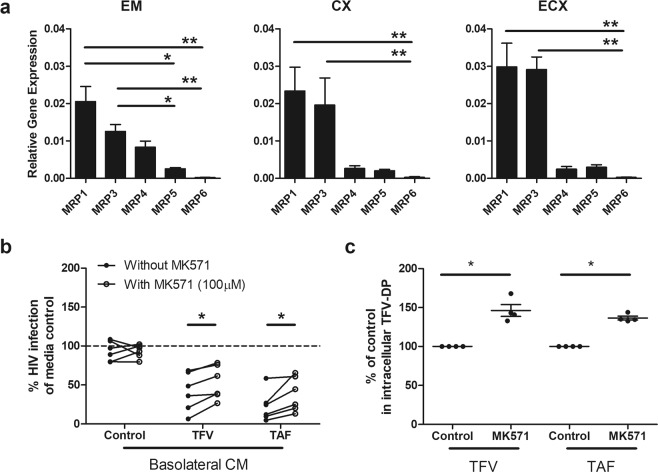
Blockade of Multidrug resistance-associated proteins (MRP) leads in part to decreased anti-HIV activity in basolateral epithelial CM via increased intracellular accumulation of TFV-DP. **(a**) MRP (1, 3, 4, 5 and 6) gene expression in epithelial cells from endometrium (EM), endocervix (CX) and ectocervix (ECX). Gene expression was determined by real time RT-PCR and normalized to β-Actin using epithelial cells from 4 patients with matched EM, CX and ECX. Y axis represents gene expression relative to the house keeping gene β-actin. Columns and horizontal lines represent the mean and SEM respectively. *p < 0.05. **p < 0.01. (**b**) Blockade of MRP transporter function decreases the release of TFV and TAF into the extracellular environment. EM epithelial cells were incubated with TFV (3277 µM) or TAF (10 µM) for 20 hr, followed by the addition of a human MRP-specific inhibitor MK571 (100 µM) to both the apical and basolateral compartments for 4 hr. Following washout, fresh media +/− MK571 (100 uM) was added to both compartments for an additional 24 hr after which the basolateral CM was collected. Activated CD4+ T cells from blood were incubated with basolateral CM for 24 hr prior to HIV infection (Methods). Data were normalized to % of HIV infection of CD4+ T cells infected in the absence of basolateral CM (media control). Each circle represents a different individual patient (n = 6) with dark circles representing basolateral CM collected from cells not treated with MK571, and open circles basolateral CM from cells treated with MK571 Data are normalized to the infection of CD4+ T cells in the absence of CM (media control) and set to 100%. *p < 0.05. (**c**) Intracellular TFV-DP levels in purified polarized EM epithelial cells increase following incubation with TFV (3277 µM) or TAF (10 µM) for 24 hr in the presence of MK571 (100 µM). Data were normalized to % of intracellular TFV-DP without MK571 in experiments using EM epithelial cells from 4 individual patients. Each circle represents a different patient. Horizontal lines represent the mean and SEM respectively. *p < 0.05.

Our finding of preferential basolateral release by epithelial cells of ARVs at concentrations capable of protecting underlying cells from infection, suggested that ARV release may in part be mediated through active transport. MRP1 and MRP3 have been reported to be selectively expressed in the basolateral surface of intestinal epithelial cells and mediate TFV transport outside the cell^40^. To determine whether primary FRT epithelial cells contain MRPs, polarized epithelial cells from the EM, CX and ECX were analyzed for the presence of MRP1, MRP3, MRP4, MRP5 and MRP6. As seen in Fig. 5a, epithelial cells from all 3 sites expressed MRPs, with MRP1 and MRP3 expressed at significantly higher levels than MRP4, MRP5 and MRP6.

Based on these findings, we hypothesized that MRP transporters might in part be responsible for the preferential basolateral release of TFV and TAF from polarized epithelial cells. To test this, EM epithelial cells were incubated with TFV or TAF for 20 hr after which the human MRP-specific inhibitor MK571 was added to apical and basolateral compartments for 4 hr^41^. MK571 was present in wash media and during the following 24 hr when cells were placed in fresh media. If MRPs are responsible for basolateral transport of TFV, we would expect that after MRP blockade less TFV would be released into the CM resulting in increased infection of CD4+ T cells by HIV. As see in Fig. 5b, MK571 partially reversed the protective effect of basolateral CM from ARV-treated epithelial cells, for both TFV and TAF by increasing HIV infection, but had no effect on viral infection in the absence of ARVs. To test if this effect could be maximized, MK571 was added at the start of incubation along with TFV or TAF for 24 hr prior to the collection of CM after washing cells and further incubation. We found that extended exposure did not further inhibit ARV release beyond that seen when MK571 was added for the last 4 hrs of culture (not shown).

To confirm that MK571 was biologically active, we measured intracellular TFV-DP levels in epithelial cells incubated with TFV and TAF for 24 hr in the presence of MK571. As seen in Fig. 5c, MK571 treatment resulted in a modest but significant increase in intracellular TFV-DP in epithelial cells. This increase in TFV-DP correlated with the loss of HIV protection when CD4+ T cells were incubated with basolateral CM from ARV-treated epithelial cells, suggesting a decreased efflux of TFV from epithelial cells. Overall, these findings indicate that MRPs contribute partially to the basolateral release of TFV in the direction of stromal tissues.

## Discussion

Overall, our studies demonstrate that following incubation with ARVs, EM, CX and ECX epithelial cells and fibroblasts retain and subsequently release TFV or TAF that provide downstream protection of CD4+ T cells from HIV acquisition. ARV release by polarized epithelial cells is selective towards the subepithelial (basolateral) compartment, where it can be taken up by underlying fibroblasts. Together, both epithelial cells and fibroblasts gradually release ARVs to protect CD4+ T cells. Following apical TFV or TAF administration, there is complete protection from HIV infection for the first 24 hr. However, after drug removal, epithelial cells and fibroblasts act as tissue reservoirs that slowly release TFV or TAF, conferring partial protection that persists for at least three days. These findings highlight a previously un-recognized role of the mucosal environment in protection against HIV acquisition by modulating availability of ARVs to HIV-target cells.

Previous studies using purified cells from the FRT to measure intracellular TFV-DP following incubation with TFV demonstrated that epithelial cells and fibroblast TFV-DP concentrations were 100- and 10-fold higher respectively than those found in CD4+ T cells^24^. These findings prompted us to ask whether epithelial cells and fibroblast accumulation of ARVs extends protection by acting as a reservoir, or compromises it by reducing drug availability to HIV target cells in the FRT. A major finding in the present study is that by gradually releasing TFV and TAF at concentrations sufficient to provide protection of CD4+ T cells from HIV infection, epithelial cells and fibroblasts extend ARV-mediated protection by providing a reservoir that lengthens the effectiveness of TFV and TAF beyond their initial application. Recognizing that ARVs can be administered orally or locally in the FRT, other studies have reported that the route of administration (oral vs. vaginal) influences the time needed for TFV to reach protective levels within HIV-target cells^31^. Following a single oral dose of TFV, the concentration of TFV and TFV-DP in the gastrointestinal (GI) mucosa is 100-fold greater than the FRT, and requires 7–14 days to reach a protective concentration in the FRT^32^. A possible reason for this delay could be that orally administered ARVs must GI cross the mucosa prior to entering circulation. Our studies indicating that TFV accumulates in FRT epithelial cells and fibroblasts, suggest that if a comparable concentration occurs in the GI tract cells, it may delay ARV levels reaching effective concentrations in the FRT. This is particularly relevant for TAF which was undetectable in most FRT tissues following a single oral dose, while still being detectable in circulation^42^. However, by acting as a local ARV reservoir, GI epithelial cells would also extend ARV protection for GI CD4+ T cells, which are susceptible to HIV infection. Whether FRT epithelial cells, and those from other mucosal sites, can act as a reservoir for other ARVs being considered for PrEP, such as dapivirine, remains to be determined, but could be an important factor in modulating their efficacy.

An important finding is that ARVs released by epithelial cells can be taken up and re-released by underlying fibroblasts. This suggests that *in vivo*, epithelial cells and fibroblasts work in tandem, with each contributing to a sustained tissue concentration that provides extended protection of HIV target cells. Although we did not measure the concentrations of TFV or TAF being released, based on our previous *in vitro* studies^8^, we know that the concentrations of TFV and TAF that result in partial protection of activated blood CD4+ T cells, as seen in this study, are very low (1–5 μM and 5–10 nM for TFV and TAF respectively) and several logs below the detectable limits for measuring TFV and TAF^7,24^. Moreover, apical concentrations were below these concentrations (1–5 μM and 5–10 nM for TFV and TAF respectively), since we repeatedly found no evidence of protection. However, *in vivo*, tissue protection may be greater than predicted in our *in vitro* studies, because of the limited fluid volume within the interstitial space of the FRT, where concentrations of ARVs would be expected to be several folds higher than in our system. This conclusion is based on recent findings demonstrating that, whereas the stroma was thought to be dense connective tissue, the interstitium at mucosal sites is now recognized as a series of fluid-filled compartments^43^. Given the partial protection we observe with CM, we speculate that protection *in vivo* would be more complete, given the reduced interstitial volume and consequently higher concentration of secreted ARVs. Further studies are needed to obtain a more accurate profile of *in vivo* intracellular and interstitial concentrations of ARVs in FRT tissues, recognizing that even within the same tissue, drug distribution, and therefore efficacy, is likely to vary with cell type.

**Figure 6:**
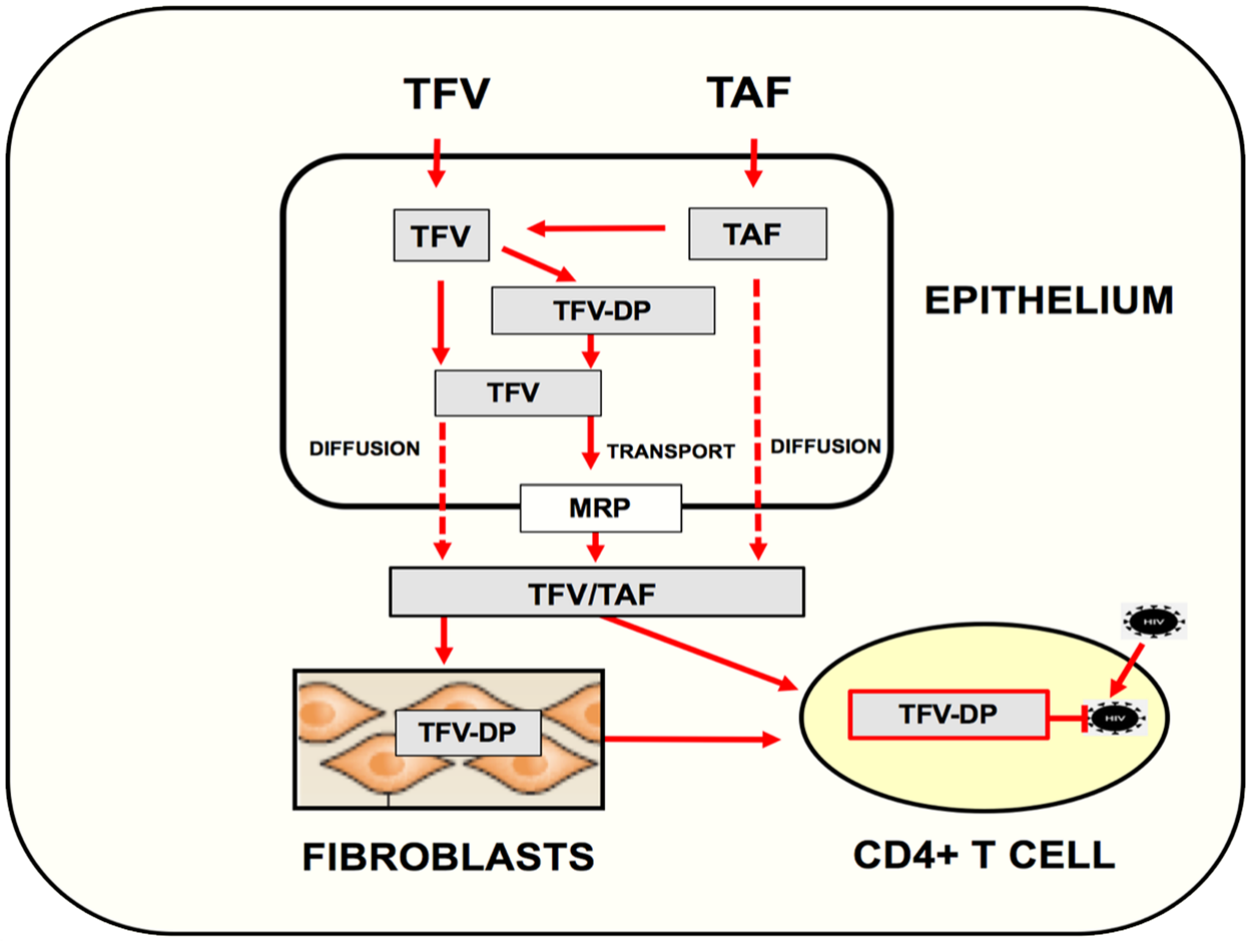
Proposed pathway of TFV and TAF mediated protection of CD4+ T cells by epithelial cells and fibroblasts in the female reproductive tract. TFV and TAF readily enter epithelial cells throughout the female reproductive tract (FRT) where they are converted to TFV-DP, the active metabolite that inhibits HIV replication. Intracellular TFV-DP can be converted back into TFV. While movement of TFV-DP out of the cell is restricted, TFV and TAF are released basolaterally into the extracellular environment both by diffusion across the plasma membrane and/or active transport out of cells by MRPs expressed on the cell surface. Once released to the underlying stroma, TFV/TAF either act directly on CD4+ T cells to prevent HIV infection or are taken up by stromal fibroblasts, which similar to epithelial cells, gradually release TFV/TAF to the local environment. In doing so, both epithelial cells and stromal fibroblasts function as reservoirs that extend the time interval of protection beyond the initial uptake of TFV/TAF by mucosal CD4+ T cells, the primary targets of HIV infection.

An unexpected finding in our study was that whereas basolateral secretions from TFV and TAF treated epithelial cells reduced HIV infection of CD4+ T cells, apical secretions from the same cells had no effect. The reasons for this unique phenotype are unclear but previous studies have shown that FRT epithelial cells can selectively secrete cytokines and chemokines via their apical or basolateral membranes^35,44,45^. To explore the underlying mechanisms responsible for basolateral release, we examined the expression of MRPs, which eliminate drugs from the cells, and found that FRT epithelial cells express MRP 1, 3, 4, 5 and 6. Chemical inhibition of MRP function resulted in a moderate but significant increase in intracellular TFV-DP levels in epithelial cells, and a decrease in CD4+ T cell protection by basolateral secretions from both TAF and TFV pre-loaded epithelial cells. These findings are consistent with increased TFV and TAF accumulation inside of epithelial cells when transporters are inhibited and suggest that MRPs are selectively expressed on the basolateral membrane of epithelial cells^40^. However, since protection was only partially reduced, this raises questions about the extent to which other transporters may also be involved in TFV and TAF efflux, since different families of transporters have been described in FRT cells, including BCRP and P-gp^27^. Another potential explanation for the partial effect seen in these studies is that initial levels of TFV and TAF in epithelial cells following preloading diffuse out and mask transporter function as ARVs exit the cell (Fig. 6). Alternatively, since MRPs are a family of anionic transporters^46^, some of these transporters may be insensitive to the inhibitor used in our studies. Additional mechanisms that mediate the selective transport of TFV from FRT epithelial cells remain to be elucidated.

An interesting finding in our study was that TFV and TAF were differentially taken up by epithelial cells when loaded apically (Fig. 2f). Previous studies have demonstrated that the Organic Anion Transporter (OAT) proteins OAT1 and 3 are capable of transporting tenofovir into the cells lining the kidney proximal tubule^20,47^. Other studies also demonstrated that endothelial cells express OAT1 and OAT3 receptors, however, we and others have previously measured these receptors and failed to detect their presence in primary epithelial cells and fibroblasts from throughout the FRT^18,36^. Therefore, additional transporters other than OAT1 and OAT3 may be involved in the differential apical uptake between TFV and TAF. Others have demonstrated that TFV uptake was partially mediated through endocytosis in vaginal epithelial cell lines and T cells, while TFV disoproxil fumarate entered these cells by passive diffusion^18^. Whether this same mechanism may explain the difference in apical uptake between TFV and TAF by primary epithelial cells throughout the FRT remains to be determined. Further studies are needed to identify potential additional transporters and establish their role in the sustained HIV protection we report in this paper.

Our findings of preferential basolateral release of ARVs have important clinical implications. First, as shown by others^48^, when administered intravaginally to women, TFV in cervical vaginal lavages (CVL) decreases by 10–100 fold within 24 hr. When women have sex during this time, TFV concentrations further decrease in the vaginal lumen. These findings further support our conclusion that epithelial cells and fibroblasts serve as important sources of ARVs that are gradually released into underlying tissues to provide protection over a period of days. Second, it implies that TFV concentrations in CVL following topical or oral administration of TFV and TAF are not a good indicator of tissue concentrations and protection, and should not be used to monitor adherence or anti-HIV activity in clinical trials. Third, our findings offer another potential explanation for why TFV did not protect women with genital inflammation derived from STIs^49^, which probably increases target cell presence in the mucosa, and induces epithelial injury in the form of crypt abscesses, mucosal erosions and ulceration. Under these conditions immune cells would not be protected once the initial drug dose is washed out. Given our results, different factors that alter TFV-DP concentrations in epithelial cells and fibroblasts, such as inflammation^49^, sex hormones such as estradiol which is known to increase uterine epithelial cell intracellular TFV-DP concentrations^24^, or menopause which lowers concentrations^29,50^, may be more relevant for protection than previously anticipated.

Beyond dosage, the efficacy of an ARV is modulated by several factors including local inflammation, use of contraceptives, composition of the microbiome, and presence of sexually-transmitted infections^8,51–53^. Our previous studies have demonstrated that medroxyprogesterone acetate (MPA), a commonly used contraceptive in Sub-Saharan Africa associated with increased risk of HIV acquisition, inhibits the conversion of TFV to TFV-DP in activated blood CD4+ T cells at concentrations similar to that used in this study, thus leading to decreased protection against HIV infection^8^. Similarly, MPA inhibits conversion of TAF to TFV-DP in FRT CD4+ T cells^8^. Alterations in the vaginal microbiome are also linked to decreased ARV efficacy. Anaerobic bacteria such as *Gardnerella vaginalis* commonly seen in bacterial vaginosis, decreased the pool of vaginal TFV available for HIV target cells faster than *Lactobacillus* species^51^. Lastly inflammation, either due to the composition of vaginal gels^54^ or pre-existing sexually-transmitted infections^53^, can drive the recruitment of target cells to the mucosal surface bringing them into proximity of incoming HIV, which in turn can undermine the effectiveness of TFV^49^. Our studies add an additional component to the complexity of interactions that dictate ARV effectiveness. Our results of preferential basolateral release of ARVs suggest that TFV or TAF treatment would not protect against HIV infection events happening on the mucosal surface, induce under conditions of inflammation or STIs that attract target cells to the surface.

While there is evidence that TFV is released basolaterally by intestinal (Caco-2) and endometrial (HEC-1-A) epithelial cell lines, and that TFV is present in *ex vivo* treated tissue explants^55^, our study is novel in several aspects. First, ours is the first study to demonstrate that primary epithelial cells throughout the FRT accumulate TFV-DP inside the cells and, after the initial drug inoculum is cleared, gradually release TFV, and release it in sufficient amounts and in a sustained manner to protect CD4+ T cells from HIV infection. Second, we demonstrate for the first time that the same is true for TAF, and that the same effect can be achieved with much lower doses than TFV. Third, while previous studies have demonstrated the presence of TFV in the basolateral compartment, to the best of our knowledge, no formal comparison of basolateral and apical release has been performed until now. Lastly, our findings are unique in that they demonstrate the release of ARVs (TFV and TAF) by both columnar (EM, CX) and squamous (ECX) EC and underlying stromal fibroblasts from each of these sites.

As seen in our summary (Fig. 6), on entering cells TFV is converted to TFV-DP, its biologically active form, which is restricted to the intracellular environment on immune and non-immune cells^7,8,24^. Similarly, TAF enters cells and is sequentially converted to TFV, and then to TFV-DP^20^. As a part of this metabolic cycle, TFV-DP from TFV and TAF is converted back into TFV, which either leaves the cell or is metabolized to adenosine^20^. Our studies suggest that following washout, two distinct steps (diffusion and transport) regulate the efflux of TFV and possibly TAF and are involved in the protection of CD4+ T cells from HIV infection. We speculate that basolateral protection following ARV washout, is due to the gradual release of TFV and TAF that is retained inside the epithelial cells and fibroblasts as well as TFV-DP and TFV-MP until converted back to TFV. Upon efflux and uptake by CD4+ T cells, TFV is converted back to TFV-DP which exerts a protective effect by inhibiting viral replication. The gradual conversion of TFV-DP and release of TFV from epithelial cells and stromal fibroblasts steadily decreases over days following washout, and thus the protective effect against HIV infection also decreases. Also shown is the transfer of TFV from epithelial cells to stromal fibroblasts and the fibroblasts ability to gradually release TFV to protect CD4+ T cells from HIV infection. Further studies are needed to more fully define the mechanisms whereby epithelial cells and fibroblasts throughout the female reproductive tract function as reservoirs to extend protection of CD4+ T cells beyond the initial ARV tissue exposure.

In conclusion, our results demonstrate that epithelial cells and underlying stromal fibroblasts from throughout the FRT readily take up TFV and TAF and produce TFV-DP. After initial drug exposure, degradation of TFV-DP back to TFV as well as any residual TFV and TAF can be gradually released to confer protection of CD4+ T cells from HIV infection for several days. Our findings demonstrate a previously unrecognized role of the mucosal environment in controlling ARV-mediated protection against HIV acquisition and suggest that factors that modify retention of ARVs, and intracellular concentrations of their active metabolites, such as TFV-DP, in epithelial cells and fibroblasts may be more relevant for protection than previously anticipated.

## Supplementary information

Supplementary Information File
