# Supplementary material for: Epithelial Cells and Fibroblasts from the Human Female Reproductive Tract Accumulate and Release TFV and TAF to Sustain Inhibition of HIV Infection of CD4+ T cells
Source: Sci Rep. 2019 Feb 12;9:1864. doi: 10.1038/s41598-018-38205-y (PMC6372694; doi:10.1038/s41598-018-38205-y)
Supplement: Supplementary file 1 — Supplementary Information File [file 41598_2018_38205_MOESM1_ESM.pdf]

Epithelial Cells and Fibroblasts from the Human Female Reproductive Tract Accumulate and Release TFV and TAF to Sustain Inhibition of HIV Infection of CD4+ T cells

Zheng Shen, Marta Rodriguez-Garcia, Mickey V. Patel, Jack Bodwell,

and Charles R. Wira

Supplementary Figure 1. (a) Correlation between HIV infection of CD4+ T cells treated with basolateral epithelial CM and intracellular TFV-DP levels in EM epithelial cells following TFV (Figure 2b) and TAF (2c) incubation as described in Fig. 2. Vertical bars indicate mean +/- SE of HIV infection. Horizontal bars indicate the mean +/- SE of TFV-DP values. **(b)** Real-time reverse transcription-polymerase chain reaction (RT-PCR) was done to measure the effect of TFV on 4 epithelial cell tight junction genes TJP1, OCLD, CLDN2 and CLDN4. Incubation of cells with TFV (3277μM) had no effect on tight junction gene expression.

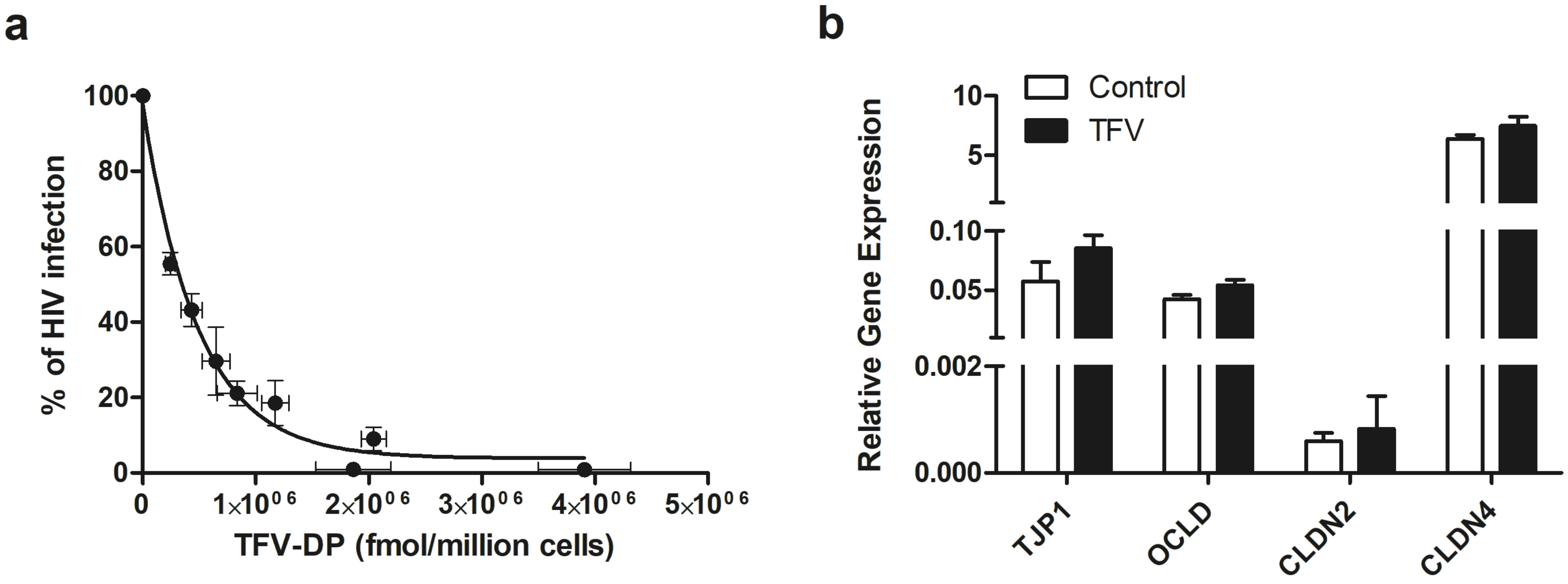
a
100
80
60
40
20
0
% of HIV infection
0
1×10⁰ 6
2×10⁰ 6
3×10⁰ 6
4×10⁰ 6
5×10⁰ 6
TFV-DP (fmol/million cells)
b
Control
TFV
10
5
0.10
0.05
0.002
0.000
Relative Gene Expression
TJP1
OCLD
CLDN2
CLDN4
